# LINC00665 Facilitates the Malignant Processes of Osteosarcoma by Increasing the RAP1B Expression via Sponging miR-708 and miR-142-5p

**DOI:** 10.1155/2021/5525711

**Published:** 2021-07-07

**Authors:** Limin Wang, Xinghua Song, Lijun Yu, Bing Liu, Jinfeng Ma, Weihua Yang

**Affiliations:** ^1^Department of Orthopedics, Dongying Traditional Chinese Medicine Hospital, Dongying, 257055 Shandong, China; ^2^Department of Clinical Laboratory, Dongying Traditional Chinese Medicine Hospital, Dongying, 257055 Shandong, China; ^3^Department of Orthopedics, Affiliated Hospital of Qingdao University, Qingdao 266003, China; ^4^Department of Surgery, Dongying Traditional Chinese Medicine Hospital, Dongying, 257055 Shandong, China

## Abstract

Osteosarcoma (OS) is a kind of fatal primary bone tumors in adolescents and young adults. Long noncoding RNAs (lncRNAs) are a group of noncoding RNAs which occupy a part of the latest hot topics. We aimed to investigate the roles of lncRNA LINC00665 in OS in this study. In this study, we found that LINC00665 was highly expressed in OS tissues and cell lines, and its high expression was associated with malignant feature and poor prognosis of OS. In OS cells, LINC00665 could facilitate the proliferation, migration, and invasion to play an oncogenic role. Mechanistically, LINC00665 served as a sponge for miR-708 and miR-142-5p and positively mediated the expression of their target RAP1B. Finally, we confirmed that LINC00665 exercised its biological functions by mediating RAP1B. In conclusion, LINC00665 is overexpressed in OS and facilitates the malignant processes of OS cells by increasing the RAP1B expression via sponging miR-708 and miR-142-5p.

## 1. Introduction

Osteosarcoma (OS) is one of the most frequently occurring primary bone tumors in adolescents and young adults [1, 2]. Although combination of multiple therapies prolonged the life expectancy of majority patients, the 5-year survival rate of OS is still low due to its high malignancy [3]. Many tumor-related molecules play key parts in the tumorigenesis of OS, making the pathogenesis of OS awfully complex [4]. Thus, it is urgently needed to find these molecules and uncover their roles in OS.

Long noncoding RNAs (lncRNAs) are a group of noncoding RNAs with over 200 nucleotides in length and have no protein-coding ability [5]. They are involved in a wide array of physiological and biological cellular processes including transcriptional regulation, epigenetic modification, and acting as microRNA (miRNA) sponges [6–8]. A large number of lncRNAs have been found to be abnormally expressed in multiple tumors, exerting great impacts on carcinogenesis and cancer progression [9]. Regarding OS, lncRNAs have been identified as oncogenic or tumor suppressive genes to participate in the carcinogenesis or antitumor process [10]. For instance, lncRNA CEBPA-AS1 is found to be weakly expressed in OS and inhibit proliferation and migration and stimulate apoptosis of OS cells [11]. LncRNA RP11-361F15.2 is significantly increased in OS and promotes osteosarcoma tumorigenesis by inhibiting M2-Like polarization of tumor-associated macrophages of CPEB4 [12].

Long intergenic noncoding RNA 00665 (LINC00665), a newly identified lncRNA transcribed from chromosome 19q13.12, has been reported to function as an oncogene in several cancers such as prostate cancer [13], breast cancer [14], gastric cancer [15], non-small-cell lung cancer [16], and hepatocellular carcinoma [17]. To our knowledge, the expression and functions of LINC00665 in OS have not been investigated previously. Hence, in this study, we intended to explore the expression, biological function, and possible mechanisms in OS.

## 2. Materials and Methods

### 2.1. Tissue Samples

Paired OS and the adjacent nontumorous normal tissues were obtained from 42 patients who underwent complete resection surgery at the Dongying Traditional Chinese Medicine Hospital. The adjacent normal tissues were at least 4 cm away from the tumor tissues, and all the tissues were histologically characterized by pathologists. None of these patients received radiotherapy or chemotherapy before surgery. The present study was approved by the ethics committee of Dongying Traditional Chinese Medicine Hospital and strictly followed the Declaration of Helsinki. Written informed consents were provided by all participants.

### 2.2. Cell Culture and Transfection

OS cell lines (MG63, U2OS, 143B and Saos-2) and normal osteoblast cell line (hFOB) were obtained from American Type Culture Collection (ATCC, USA). These cell lines were maintained in DMEM medium (Thermo Fisher Scientific, USA) with 10% FBS (Gibco, USA) in a humidified atmosphere of 5% CO_2_ at 37°C. Cell lines used in this study had been authenticated by STR cell identification from May 2016 to April 2018. We confirm that all experiments were performed with mycoplasma-free cells. Cell transfection was carried out with Lipofectamine 3000 (Invitrogen, USA). The full-length LINC00665 was inserted into a pcDNA3.1 vector to realize the endogenous LINC00665 expression, and short-hairpin RNA (shRNA) targeting LINC00665 was constructed to knockdown LINC00665.

### 2.3. Real-Time Quantification Polymerase Chain Reaction (qRT-PCR)

Total RNAs were extracted from tissues or cells using TRIzol Reagent (Invitrogen). Nuclear and cytoplasmic RNAs of OS cells were separated by a PARIS Kit (Invitrogen, USA). RNAs were then reversely transcribed by a PrimeScript RT Master Mix (Perfect Real Time) (TaKaRa, Japan). SYBR Green Realtime PCR Master Mix (Takara, Japan) and an ABI7500 system were utilized to accomplish qPCR reaction. Relative RNA expressions were calculated by the 2^−*ΔΔ*CT^ method and normalized to GAPDH (for LINC00665) or U6 (for miRNAs). Sequences of primers used in this study were LINC00665, forward, 5′-GGTGCAAAGTGGGAAGTGTG-3′, reverse, 5′-CGGTGGACGGATGAGAAACG-3′; miR-708, forward, 5′-GGCGCGCAAGGAGCTTACAATC-3′, reverse, 5′-GTGCAGGGTCCGAGGTAT-3′; miR-142-5p, forward, 5′-AGCTCGCGCATAAAGTAGAAAG-3′, reverse, 5′-TATGGTTGTTCTCGTCTCTGTGTC-3′; GAPDH, forward, 5′-ACCACAGTCCATGCCATCAC-3′, reverse, 5′-TCCACCCTGTTGCTGTA-3′; U6, forward, 5′-GCTTCGGCAGCACATATACTAAAAT-3′, reverse, 5′-CGCTTCACGAATTTGCGTGTCAT-3′.

### 2.4. Cell Proliferation Assays

The proliferation of OS cells was investigated by the cell counting kit-8 (CCK8) assay and 5-ethynyl-2′-deoxyuridine (EdU) assay. For the CCK8 assay, cells were seeded into a 96-well plates and transfected with plasmids or oligonucleotides. CCK-8 reagent (Dojindo, Japan) was added into each well after cells were grown for 24, 48, and 72 hours. After incubation, optical density at 450 nm was detected using a microplate reader. For the EdU assay, cells were seeded in a 96-well plate and transfected for 48 h. Cell-light™ EdU ApolloR567 in Vitro Imaging Kit (Ribobio, China) was applied to fluorescently label cells those were synthesizing DNA. Images were taken with a fluorescent microscope to calculate the EdU-positive cells.

### 2.5. Cell Migration and Invasion Assays

Cell migration capability was assessed by the wound-healing assay. Transfected cells were inoculated in 6-well plates for 24 hours and scratched with a pipette tip (0 h). Cells were then cultured in FBS-free DMEM medium for another 24 hours (24 h). Cell migration was observed under the microscope.

Cell invasion potential was evaluated by the transwell assay using transwell chambers (Corning, USA). Cells transfected for 48 h were harvested and seeded in the matrigel coated upper chamber with FBS-free DMEM medium. The lower chamber was added with DMEM medium supplemented with 10% FBS. Cells that pierced through the membrane were fixed and dyed to count numbers.

### 2.6. Fluorescence In Situ Hybridization (FISH) Assay

The FISH assay was performed using a Fluorescent In Situ Hybridization Kit (RiboBio). Fluorescence-conjugated probes for LINC00665, miR-708, and miR-142-5p were designed and synthesized by RiboBio. There probes were used to fluorescently locate LINC00665, miR-708, and miR-142-5p, and DAPI served to show nucleus.

### 2.7. RNA Immunoprecipitation (RIP) Assay

The RIP assay was conducted using a Magna RIP RNA-binding protein immunoprecipitation kit (Millipore, USA). Cells were lysed with lysis buffer and incubated with magnetic beads coated with Ago2 antibody or IgG antibody. The RNA level in the immunoprecipitate complex was analyzed by qRT-PCR.

### 2.8. Luciferase Reporter Assay

The wild-type (wt) miRNAs target sites in LINC00665 or RAP1B 3′UTR, and the mutant-type (mut) binding sites were synthesized and cloned into the downstream of a pmirGLO vector (Promega, USA). Cells were cotransfected with pmirGLO luciferase plasmid and miRNA mimics. A Dual-Luciferase Reporter Assay System (Promega, USA) was used to detect the luciferase activity.

### 2.9. Western Blot Assay

Total proteins were isolated by RIPA buffer containing protease inhibitor. Proteins were separated on the 10% sodium dodecyl sulfate–polyacrylamide gradient gel and then transferred onto PVDF membranes (Millipore). The blocked membranes were incubated with primary antibodies (Anti-RAP1B, ab182606, Abcam, USA) at 4°C overnight and secondary antibody at room temperature for 1 h. Signals were visualized using ECL Substrates (Millipore), and GAPDH was used as an internal control.

### 2.10. Statistical Analysis

Statistical analyses were implemented with SPSS 19.0 software (IBM, USA). All data from at least three independent experiments were presented in terms of the mean ± standard deviation (SD). Differences between two or more groups were evaluated by Student's *t*-test or one-way analysis of variance (ANOVA), respectively. *P* < 0.05 was considered to be statistically significant.

## 3. Results

### 3.1. LINC00665 Is Upregulated in OS

**Figure 1:**
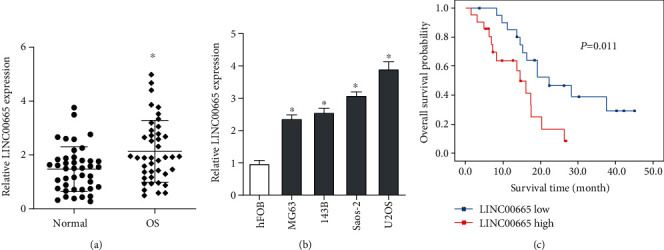
LINC00665 is upregulated in OS. (a) Expressions of LINC00665 in paired OS and nontumorous normal tissues were detected by qRT-PCR. (b) Expressions of LINC00665 in OS cell lines and normal osteoblast cell line were detected by qRT-PCR. (c) Overall survival of OS patients was analyzed by the Kaplan-Meier method. ^∗^*P* < 0.05.

First, qRT-PCR was utilized to determine the LINC00665 expression in OS tissues and cell lines. We observed that the expression of LINC00665 was increased in OS tissues (Figure 1(a)) and cell lines (Figure 1(b)). After evaluating the correlation between the LINC00665 expression and clinicopathological characteristics, we noticed that patients with larger tumor size and later clinical stages presented higher LINC00665 level (Table 1). We also exhibited that high LINC00665 expression implied poor overall survival of OS patients (Figure 1(c)).

### 3.2. LINC00665 Facilitates the Malignant Progressions of OS Cells

**Figure 2:**
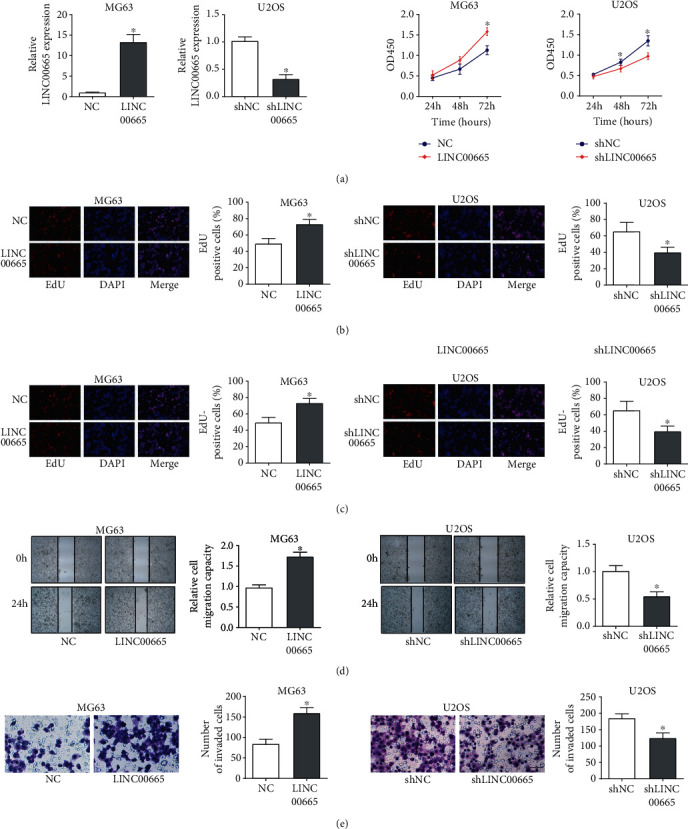
LINC00665 facilitates the malignant progressions of OS cells. (a) Overexpression or knockdown efficiency of LINC00665 in OS cells was determined by RT-qPCR analysis. (b, c) The impact of the LINC00665 overexpression or knockdown on OS cells proliferation was examined by CCK-8 assays (b) and EdU assays (c). (d, e) The effects of LINC00665 overexpression or knockdown on OS cells migration and invasion were assessed by wound-healing assays ((d), magnification, 40×) and transwell assay ((e), magnification, 100×). ^∗^*P* < 0.05.

To uncover the function of LINC00665 in OS, LINC00665 was endogenously overexpressed in MG63 cells and knocked down in U2OS cells (Figure 2(a)). Then, data of CCK8 assays and EdU assays showed that the overexpression of LINC00665 promoted the proliferation ability of MG63 cells, and knockdown of LINC00665 restrained the proliferation capacity of U2OS cells (Figures 2(b) and 2(c)). Moreover, the wound-healing assay and transwell assay exhibited that the overexpression of LINC00665 was raised while silence of LINC00665 depressed migration and invasion capacity of OS cells (Figures 2(d) and 2(e)). Here, we concluded that LINC00665 facilitated the malignant progressions of OS cells.

### 3.3. LINC00665 Operates as a miRNA Sponge for miR-708 and miR-142-5p

**Figure 3:**
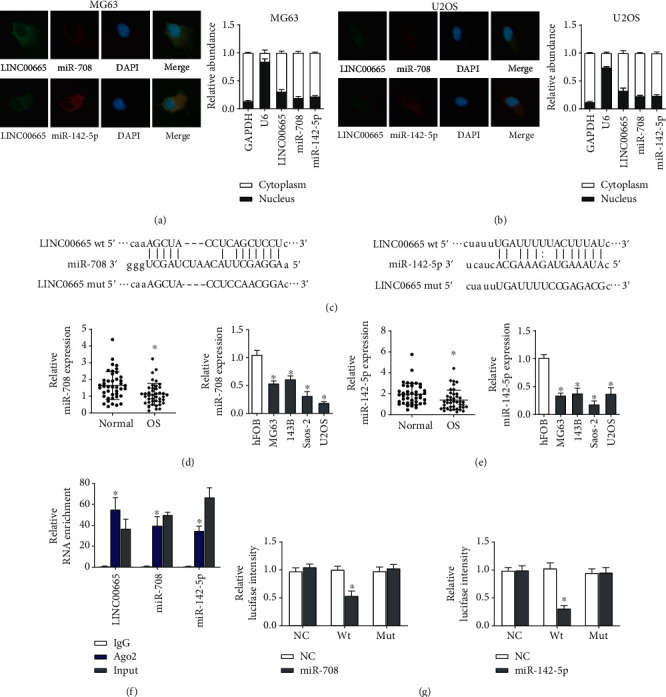
LINC00665 operates as a miRNA sponge for miR-708 and miR-142-5p. (a, b) FISH assays and qRT-PCR assays determined the subcellular localization of LINC00665, miR-708, and miR-142-5p in OS cells. (c) Predicted binding sequences of miR-708 or miR-142-5p with LINC00665. (d, e) Expressions of miR-708 and miR-142-5p in OS tissues and cell lines detected by qRT-PCR. (f) RIP assays identified the enrichment of LINC00665, miR-708, and miR-142-5p in Ago2 conjugates. (g) Predicted binding sequences of miR-708 or miR-142-5p with LINC00665 were validated by luciferase reporter assays. ^∗^*P* < 0.05.

To explore the molecular roles of LINC0065 in OS, we performed FISH assays and qRT-PCR assay to study the subcellular localization of LINC00665. We found that LINC00665 mainly existed in the cytoplasm of MG63 and U2OS cells (Figures 3(a) and 3(b)). LINC0065 was reported to mainly locate in cytoplasm and functioned as miRNA sponges in lung adenocarcinoma and breast cancer cells [18, 19]. So, we predicted the miRNAs that might bind to LINC00665 via the starBase database and found that miR-708 and miR-142-5p were potential candidates (Figure 3(c)). We also presented that miR-708 and miR-142-5p showed similar subcellular localization with LINC00665 (Figures 3(a) and 3(b)) and decreased expression in OS tissues and cell lines (Figures 3(d) and 3(e)). Subsequently, we carried out RIP assays, and the results revealed that LINC00665, miR-708, and miR-142-5p were significant enriched in Ago2 conjugates (Figure 3(f)) which indicated that these genes may possess a common acting basis. More important, the specific binding sites were validated by luciferase reporter assays (Figure 3(g)). These data all together suggested that LINC00665 operated as a miRNA sponge for miR-708 and miR-142-5p.

### 3.4. LINC00665 Upregulates the RAP1B Expression via miR-708 and miR-142-5p

**Figure 4:**
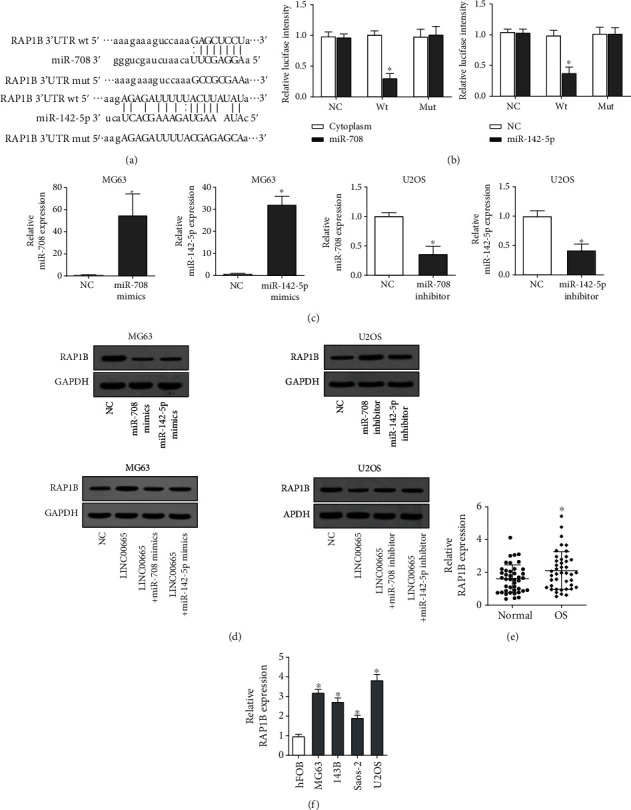
LINC00665 upregulates the RAP1B expression via miR-708 and miR-142-5p. (a) Predicted binding sequences of miR-708 or miR-142-5p with 3′UTR of RAP1B mRNA. (b) Predicted binding sequences of miR-708 or miR-142-5p with 3′UTR of RAP1B mRNA were validated by luciferase reporter assays. (c) The efficiency of the overexpression and knockdown of miR-708 and miR-142-5p in OS cells detected by qRT-PCR. (d) Western blot assays indicated the impact of miR-708 and miR-142-5p on the protein level of RAP1B, as well as the impact of LINC00665 together with miR-708 or miR-142-5p on the protein level of RAP1B. (e) Expressions of RAP1B mRNA in paired OS and nontumorous normal tissues were detected by qRT-PCR. (f) Expressions of RAP1B mRNA in OS cell lines and normal osteoblast cell line were detected by qRT-PCR.

Next, we attempted to discover the possible target genes of miR-708 and miR-142-5p. Through starBase and microRNA.org databases, we identified RAP1B as a common target of miR-708 and miR-142-5p (Figure 4(a)). We then used luciferase reporter assays verified the specific binding of miR-708 and miR-142-5p in the 3′UTR of RAP1B mRNA (Figure 4(b)). We then overexpressed and knocked down miR-708 and miR-142-5p in OS cells (Figure 4(c)) and discovered that both miR-708 and miR-142-5p could decrease the protein level of RAP1B (Figure 4(d)). What is more, we demonstrated that LINC00665 upregulated the RAP1B expression which could be attenuated by miR-708 or miR-142-5p (Figure 4(e)). Collectively, the above data suggested that LINC00665 upregulated the RAP1B expression via miR-708 and miR-142-5p. Moreover, we found that the RAP1B expression was also increased in OS tissues (Figure 4(f)) and cell lines (Figure 4(g)), which indicated that it may play carcinogenic effects.

### 3.5. LINC00665 Exercises Its Biological Functions by Mediating RAP1B

**Figure 5:**
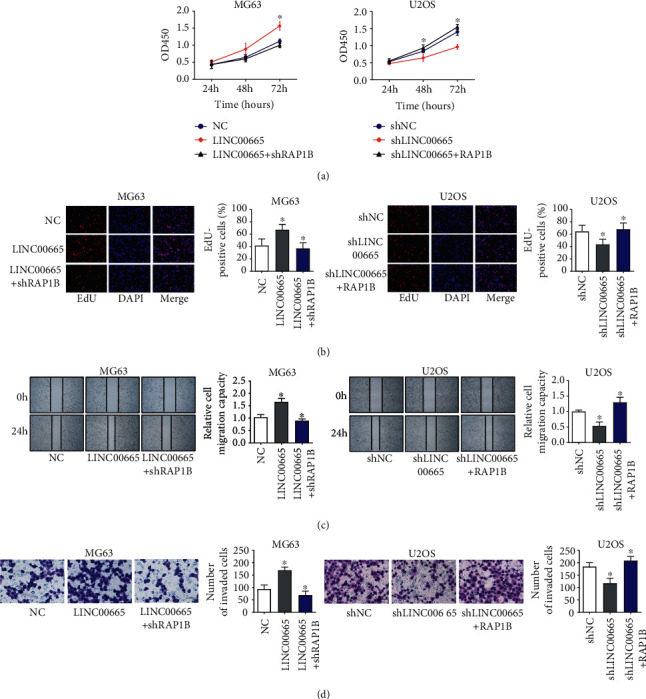
LINC00665 exercises its biological functions by mediating RAP1B. (a, b) CCK8 (a) and EdU assays (b) showed the regulation of LINC00665 and RAP1B on proliferation of OS cells. (c) Wound-healing assays showed the influence of LINC00665 and RAP1B on migration of OS cells; magnification, 40×. (d) Transwell assays displayed the influence of LINC00665 and RAP1B on invasion of OS cells, magnification, 100×. ^∗^*P* < 0.05.

Finally, we investigated the roles of RAP1B in the regulation of OS cellular progressions by LINC00665. We discovered that the RAP1B overexpression cloud absorb the accelerating effects of LINC00665 on proliferation, migration, and invasion (Figures 5(a)–5(d)). Accordingly, silence of RAP1B cloud abates the inhibitory effects of LINC00665 on proliferation, migration, and invasion (Figures 5(a)–5(d)). On the whole, these results uncovered that LINC00665 exercises its biological functions by mediating RAP1B.

## 4. Discussion

Accumulating evidence has suggested that many lncRNAs play vital roles in multiple cellular processes during tumorigenesis, whereas only few of them have been well characterized [9]. LINC00665 is a newly identified lncRNA which has been studied in several cancers but not in OS. Consistent with the conclusions of previous studies in other cancers, our present study uncovered that LINC00665 is also an oncogene in OS.

In this study, we displayed that LINC00665 was highly expressed in OS tissues and cell lines. The high LINC00665 expression was closely associated with aggressive clinicopathological characteristics and poor prognosis of OS. Therefore, as in other cancers [13–16], LINC00665 might also be a promising biomarker in OS. Functionally, we further discovered that LINC00665 facilitated OS cells proliferation, migration, and invasion with loss- and gain-of-function assays. These data means that LINC00665 also plays oncogenic roles in OS.

In addition, we showed a new regulation pathway of LINC00665 in OS. LINC00665 mainly exists in cytoplasm of OS cells that operates as a miRNA sponge for miR-708 and miR-142-5p. Then, LINC00665 upregulates the expression of RAP1B which is a target of both miR-708 and miR-142-5p in a competing endogenous RNA (ceRNA) manner. MiR-708 and miR-142-5p have been confirmed to be oncogenes in OS [20, 21]. RAP1B is a member of the RAS oncogene family and participated in multiple cellular processes including cell growth, adhesion, and differentiation [22]. Increasing evidences reveal that the deregulated activation of RAP1B is involved in a spectrum of malignancies [23]. Here, we demonstrated than RAP1B is essential for LINC00665 that exercises its biological functions of promoting proliferation, migration, and invasion.

## 5. Conclusions

In summary, we report that LINC00665 is overexpressed in OS and associated with malignant feature and poor prognosis of OS. LINC00665 facilitates the proliferation, migration, and invasion of OS cells by increasing the RAP1B expression via sponging miR-708 and miR-142-5p.

## Figures and Tables

**Table 1:** Association between clinical feathers of OS patients and LINC00665 expression in OS tissues.

Feathers	Cases	LINC00665 low	LINC00665 high	*P* value
Sex				
Male	22	10	12	
Female	20	11	9	0.537
Age(years)				
<20	25	11	14	
≥20	17	10	7	0.346
Tumor size (cm)				
<5 cm	25	16	9	
≥5 cm	17	5	12	0.028
Clinical stage				
I/IIA	23	17	6	
IB/III	19	4	15	0.001
Distant metastasis				
No	24	15	9	
Yes	18	6	12	0.061
Anatomic location				
Femur/tibia	28	13	15	
Elsewhere	14	8	6	0.513

## Data Availability

All the data is available with the handwritten notebook documented in our lab.
